# Predictors of undesirable treatment outcomes of severe acute malnutrition among inpatient children in Addis Ababa, Ethiopia: a retrospective cohort study

**DOI:** 10.1186/s12889-020-09645-x

**Published:** 2020-10-09

**Authors:** Absalat Serawit Negussie, Amare Worku Tadesse

**Affiliations:** 1 Addis Continental Institute of Public Health, P.O.box 26571/1000, Addis Ababa, Ethiopia; 2 Department of Infectious Disease Epidemiology, London School of Hygiene and Tropical Medicine, England, Keppel Street, WC1E 7HT London, UK

**Keywords:** Severe acute malnutrition, Inpatient, Undesirable treatment outcome, Children

## Abstract

**Background:**

In 2018, malnutrition contributed to 45% of all global cause of child death. These early child deaths were due to conditions that could either be prevented or treated with basic interventions. Hence, this study intended to provide a quantitative estimate of factors associated with undesirable treatment outcomes of severe acute malnutrition (SAM).

**Methods:**

We studied a retrospective cohort of 304 children aged 6–59 months with complicated SAM admitted to Yekatit 12 Hospital Medical College from 2013 to 2016. We extracted data from hospital records on nutritional status, socio-demographic factors and medical conditions during admission. The analysis was carried out using SPSS version 20. The Kaplan-Meier estimator was employed to analyze the recovery rate of the children treated for SAM and multivariable Cox regression was used to determine factors that predict inpatient undesirable treatment outcomes.

**Result:**

From a total of 304 children 6–59 months with SAM, 133 (51.4%) were boys. Marasmus was the most common type (132 (51%)) of severe acute malnutrition. The recovery, death and defaulter rate were 70.4, 12.2 and 8.2% respectively. The main predictors of undesirable treatment outcomes were found to be the presence of HIV antibody (AHR = 3.208; 95% CI: [1.045–9.846]) and sepsis (AHR = 7.677, 95% CI: [2.320–25.404]).

**Conclusion:**

The study revealed that the overall treatment outcomes were below the SPHERE standard recommendation. The main predictors of undesirable treatment outcomes among inpatient children treated for SAM were HIV and sepsis. Intervention to reduce undesirable treatment outcomes should focus on comorbidities, especially HIV and sepsis.

## Background

The United Nations’ first Millennium Development Goal (MDGs) to “eradicate extreme poverty and hunger” was measured by assessing a set of indicators, which included the prevalence of underweight children under the age of 5 years [1]. The extreme situation of malnutrition, not uncommon among children under the age of 5 years, is referred to as Severe Acute Malnutrition (SAM). The World Health Organization (WHO) defines SAM based on three anthropometric indicators: low weight for height/length ratio (WFH) below − 3 SD, presence of nutritional edema, and/ or a mid-upper arm circumference (MUAC) of less than 115 mm [2, 3]. The WHO guidelines for treatment of complicated SAM (SAM children who have clinical features of infection, metabolic disturbance, severe edema, hypothermia, vomiting, severe dehydration, severe anemia or a lack of appetite), suggests the establishment of inpatient care for the treatment and rehabilitation of severely malnourished individuals [2, 3].

Worldwide, in 2018 alone, 5.3 million children under the age of 5 years died, of which nutrition related factors contributed to about 45% of all child deaths. Children in Sub-Saharan Africa are more than 15 times likely to die before the age of 5 years than children in developed regions [4]. It is often the case that children with severe acute malnutrition have a higher risk of death from relatively common childhood illnesses such as diarrhea, pneumonia and malaria [4–6].

The United Nations Sustainable Development Goals (SDGs) are based on the MDGs which were adopted in 2015. While the MDGs were conceived particularly for developing counties, the SDGs are meant to address all UN member states and are considerably more comprehensive and ambitious than MDGs. The SDG Goal 2.2 is focused on ending all forms of malnutrition as it is noted to be the dominant cause of death among children under 5 years [3]. Ethiopia is one of the nations registered to have high child mortality rates for ages five and below. Though this mortality rate has been significantly reduced from earlier years, it is still considered high (55.2 deaths per 1000 live births in 2018) [7]. According to the 2019 Ethiopian Mini Demographic and Health Survey report, 37% of under five children were stunted, 12% severely stunted, 7% wasted, 1% severely wasted, 21% underweight and 6% severely underweight [8].

The minimum international standard set for management of SAM according to SPHERE standards is a cure rate of at least 75% and death rate less than 10% [9]. However, the case fatality rates in hospitals treating SAM in developing countries have remained high. A meta-analysis which was done in low and middle-income settings for inpatient treatment of complicated SAM shows a case fatality rate of 14% (range 5–30%) [10, 11]. There have been numerous factors attributed to high fatality cases in children admitted to inpatient care [12, 13]. Hypoglycemia, infection, anemia, dehydration, hypothermia, electrolyte imbalance, HIV and TB infection, age and sex were among the factors associated with high fatality rate of children with severe acute malnutrition [13].

Therefore, treatment for SAM with medical complications, requires further clinical insight and analysis. The intent of this study was to examine predictors of undesirable treatment outcomes of severely malnourished children hospitalized under the inpatient management scheme of SAM with medical complications.

## Method

### Study design and study setting

A retrospective cohort study was conducted at Yekatit 12 Hospital Medical College, Addis Ababa, Ethiopia. It is one of the few hospitals, among St Paul’s and Zewditu hospital, with an established nutrition therapy unit during the time of the study. Children with SAM go through an initial screening for signs of complication at out-patient department (OPD) or emergency unit. Depending on findings from the initial assessments and fulfillment of inpatient admission criteria, they would be admitted to the nutritional rehabilitation center for appropriate treatment and follow up for SAM.

Yekatit 12 Hospital Medical College uses a standardized national management protocol for management of severe acute malnutrition [14]. SAM children without co-morbidities and with good appetite would be linked to the outpatient management program. All SAM children with co-morbidities and poor appetite would be admitted to the SAM inpatient management program. After completing the inpatient management, those who satisfy the discharge criteria will be directed to community-based feeding program for further follow up [14].

### Study population, sample size and sampling technique

The study population was all SAM children aged 6–59 months who were admitted to Yekatit 12 Hospital Medical College inpatient unit between 2013 and 2016. A total of 259 children were included in this study. The following inclusion and exclusion criteria have been adopted accordingly.

#### Inclusion criteria

During the time of the study, Ethiopia has not yet adopted the current WHO recommendations for screening SAM using MUAC cut off points and admission to treatment for SAM; the Ethiopian Ministry of Health revised its MUAC cutoff point to 115 mm after 2016 and implementation with the “new” guideline started in 2017. For this reason, MUAC cutoff point used in this study was from the 2007 Ethiopian National Guideline for Management of SAM [14]. Accordingly, children within the age range of 6 months to 5 years who fulfilled the following criteria were included in the study.

1. Weight-for-height/length ratio < 70% of median or less than – 3Z- score
2. MUAC < 110 mm with Length > 65 cm
3. Presence of bilateral pitting edema with medical complications or a fail in the appetite test [14]

#### Exclusion criteria

Drop out and transfer outs were excluded in this study because their outcomes could not be traced.

The sample size was calculated using EPI info version 7.2.0.1 for a cohort study design. Based on other related studies conducted in a similar context, variables which were significantly associated with undesirable treatment outcomes were identified and used to calculate the sample size. With a 95% confidence level with 80% power and an allocation ratio of 1:1 for unexposed to exposed ratio, the computed optimal sample size (taking the largest) was 152 for each group (Table 1).

**Table 1:** Sample size calculation based on factors related to undesirable outcome in children admitted with SAM

Related Factors	CI	Power	Ratio Une:exp	Percentage Outcome in		Sample Size		
				Unexposed	Exposed	Unexposed	Exposed	Total
HIV	95%	80%	1.1	13.7%	60%	18	18	36
Gastroenteritis	95%	80%	1.1	11.1%	25.4%	115	115	230
Hypothermia(< 35 °C)	95%	80%	1.1	10.8%	33.3%	54	54	108
Sign of severe pneumonia	95%	80%	1.1	4%	21%	60	60	120
Family size	95%	80%	1.1	6.4%	16.7%	152	152	304
Blood transfusion	95%	80%	1.1	5%	27.6%	42	42	84

Data from previous studies with a focus on treatment outcome [15–17] were considered to calculate the sample size.

### Measurement

The outcome variable for this study was undesirable treatment outcomes which included death, non-respondent, and failure to respond. Socio-demographic and admission characteristics, anthropometry, type of malnutrition, comorbidities, vaccination and breast-feeding status were considered as predictor variables.

For a child admitted with SAM, the management procedure consisted of 3 phases (phase1, transition and phase 2). Children were assessed based on the Ethiopian national management protocol for SAM and in accordance with the WHO recommendations for management of SAM. The ten steps of the WHO SAM management include: treat/prevent hypoglycemia, treat/prevent hypothermia and dehydration, correct electrolyte imbalance, treat/prevent infection, correct micronutrient deficiencies, start cautious feeding, achieve catch-up growth, provide sensory stimulation and emotional support and prepare for follow-up after recovery [3, 14].

Children with SAM who either failed to regain appetite, loose edema after 4th day of admission, gain more than 5 g/kg/d after 10th day of admission or failed to gain more than 5 g/kg/d for 3 successive days during phase 2 while being on treatment, were defined as failure to respond. Children with SAM who had not reached the discharge criteria after 40 days in the inpatient unit were defined as non-responders.

Children with SAM who were discharged after fulfilling the discharge criteria (weight for height/length > 85% of median on more than one occasion or no edema for 10 days and a target weight gain during two consecutive measurements) were considered cured. Children with SAM who discontinued treatment or disappeared from nutritional rehabilitation ward before completing treatment were defined as dropouts. Children with SAM whose treatment results were unknown due to transfer to another health facility were defined as transfer outs.

### Data collection and data quality

Inpatient registry book of the Yekatit 12 Hospital Medical College which contained the admission, patient history and discharge information was used as the main data source. Medical records of children with SAM within the study time frame, from 2013 to 2016, were assessed. For eligibility. Records which were complete and that fulfilled the inclusion criteria were included in the study.

Data abstraction sheet was developed and pre-tested for its completeness and clarity.

### Data analysis

Data were entered into EPI-Info computer software program and cleaned. Data were exported to SPSS version 20.0 statistical software packages for analysis.

Descriptive statistics was used to summarize the data. Children with undesirable treatment outcomes were considered as event. The time variable was assumed to be the time to the occurrence of undesirable treatment outcome measured from admission to date of an event. Life table analysis was used to estimate the cumulative proportion of survival among children with SAM at different time point. Kaplan Meier survival curve and log-rank test was fitted to test for the presence of difference between groups of undesirable treatment outcomes and cured. Variables at *P*-value of < 0.25 in the bivariate analysis were included in the final Cox regression model to identify the independent predictors of undesirable treatment outcomes. In addition, crude and adjusted hazard ratio with their 95% confidence interval (CI) were estimated and summarized. The undesirable treatment outcomes and cure rates from this study were also compared with the minimum standard presented by the “Sphere” project.

## Result

**Fig. 1:**
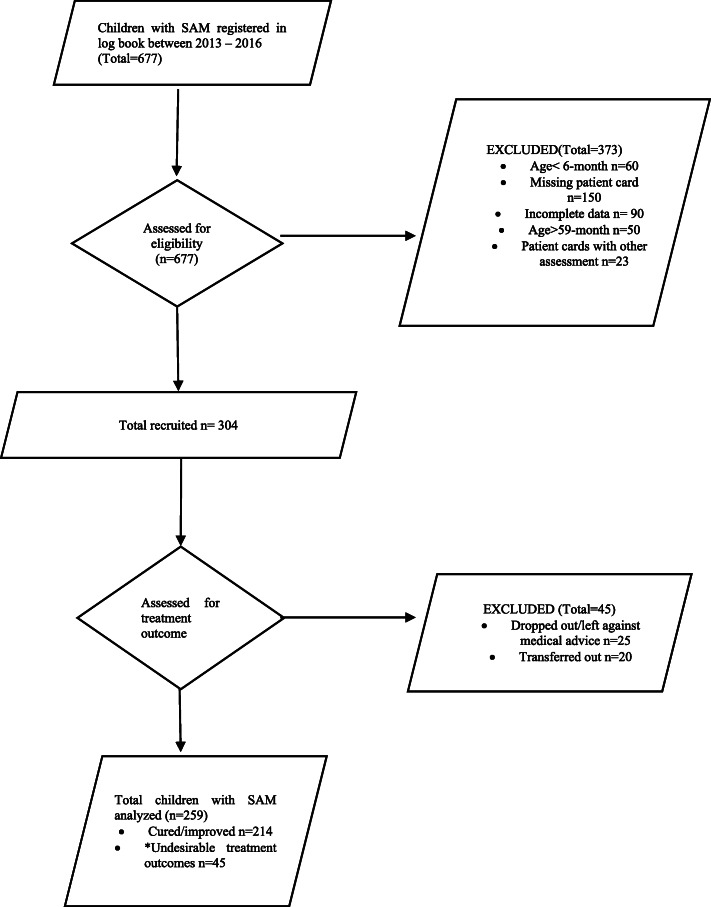
Participants flow chart. ^*^Undesirable treatment outcomes defined as death, non-respondent, or failure to respond

A total of 677 children with SAM were admitted to Yekatit 12 Hospital Medical College between 2013 to 2016; 373 were excluded from the study and while 304 were deemed eligible. Amongst these eligible cases, 25 were dropouts and 20 were transfer outs to other institutions; both groups were excluded from the analysis as their treatment outcomes could not be determined. Thus, we analyzed a total of 259 children with SAM in this study (Fig. 1). There was no significant statistical difference between background characteristics of the excluded sample and children included in this study.

### Socio-demographic characteristics, anthropometry, and type of malnutrition

From the 259 children with SAM, 133 (51.4%) were males and 126(48.6%) were females, and 214 (82.6%) were aged below 24 months with a median age of 17.5 (interquartile range: 12–24) months. Majority of the study participants (80.7%) belong to family size of less than 3 children.

With regards to the nutritional status of the children with SAM, 206 (79.5%) had a WFH ≥ 70% of median, and 160 (61.8%) had MUAC of < 11 cm and 132 (51%) had marasmus (Table 2).

**Table 2:** Socio-demography and anthropometry of children with SAM admitted to Yekatit 12 Hospital Medical College, 2013–2016

Admission characteristics	Outcome	
	Improved (%)	Undesirable outcome (%)
Socio-demographic characteristics		
Sex		
Male	114(85.7)	19(14.3)
Female	100(79.4)	26(20.6)
Age		
< 24 month	177(82.7)	37(17.3)
≥ 24 month	37(82.2)	8(17.8)
Family size		
< 3 children	173(82.8)	36(17.2)
≥ 3 children	41(82)	9(18)
Anthropometry and type of malnutrition		
WFH/L		
< 70% of median	41(77.4)	12(22.6)
≥ 70% of median	173(84)	33(16)
MUAC		
< 11 cm	130(81.2)	30(18.8)
≥ 11 cm	84(84.8)	15(15.2)
Type of malnutrition		
Non-edematous	109(82.6)	23(17.4)
Edematous	105(82.7)	22(17.3)

*WFH/L weight-for height/ or length in percent, *MUAC* mid upper arm circumference

Among SAM children with undesirable treatment outcome, 26 (20.6%) were females, 9 (18%) lived in a household with three or more children. The nutritional status of children with undesirable treatment outcome, 12 (22.6%) have WFH < 70% of median and 30(18.8%) had MUAC of < 11 cm. (Table 2).

### Clinical profile and morbidity patterns

More than half (55.5%) of children with SAM had diarrhea and a significant proportion of these children (39.6%) had pneumonia at the time of admission. Anemia, Sepsis, skin lesion (dermatitis of kwashiorkor), Tuberculosis and shock were prevalent in 67(29.5%), 19(8.4%), 20(8.8%), 18(7.9%) and 7(3.1%) among the study children respectively. HIV test was also carried out for 190(73.4%) children, of which 11(5.8%) were tested to be positive (Table 3).

**Table 3:** Clinical profile of children with SAM at admission at Yekatit 12 Hospital Medical College, 2013–2016

Admission characteristics		Outcome	
		Improved (%)	Undesirable outcome (%)
Anemia	Yes	55(82.1)	12(17.9)
Pneumonia	Yes	69(76.7)	21(23.3)
Diarrheal disease	Yes	102(81)	24(19)
Sepsis	Yes	10(52.6)	9(47.4)
Skin lesion	Yes	14(70)	6(30)
Tuberculosis	Yes	14(77.8)	4(22.1)
Shock	Yes	3(42.9)	4(57.1)
HIV Antibody	Positive	7(63.6)	4(36.4.)
Others	Yes	19(73.1)	7(26.9)

**Others:** Urinary tract infection, Electrolyte imbalance, Bacterial conjunctivitis and Otitis Media

### Treatment outcome

During the study period, 214(70.4%) of SAM children were cured, which was below the minimum recovery rate of 75% recommended in the SPHERE standard and linked to outpatient therapy. On the other hand, 37 (12.2%) had died during treatment which was also higher than the SPHERE standard recommendation of 10% mortality rate. Moreover 64.9% of the deaths occurred in the first 7 days of admission. The average (± SD) length of stay in the hospital was 16 days (±10.7), and the average weight gain during the inpatient treatment phase was 8.13 g/kg/day for non-edematous malnutrition (Table 4).

**Table 4:** Comparison of treatment outcomes with SPHERE standard indicators

Indicators	Results	^a^SPHERE standards	
		Acceptable	Alarming
Cure rate (%)	70.4%	> 75%	< 50
Death rate (%)	12.2%	< 10%	> 15
Defaulter rate (%)	8.2%	< 15%	> 25
Rate of weight gain(g/kg/day)	8.13 g/kg/day	≥ 8	< 8
Average length of stay (days)	16 days	< 30 days	

^a^SPHERE, Social and Public Health Economics Research Group

### Survival analysis

**Fig. 2:**
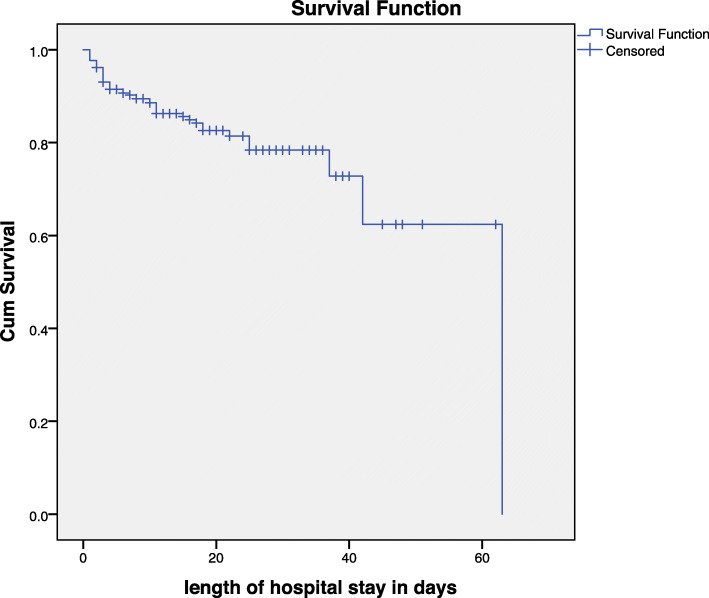
Kaplan –Meier Survival estimate among children with SAM admitted to Yekatit 12 Hospital Medical College, 2013–2016

A total of 259 children with SAM were followed for different periods with a minimum of 1 day and a maximum of 63 days with a median follow-up period of 14 days (Fig. 2). The median nutritional recovery time of the entire cohort using Kaplan Meier survival analysis is 17 days (95% CI: 15.615–18.385). The greatest number and proportion of terminal events occurred within the first 7 days (Table 5).

**Table 5:** Median nutritional recovery time of SAM during at Yekatit 12 Hospital Medical College, 2013–2016

Characteristics	Number	Median recovery time		Log rank X^2^ –value	*P*-value
		Estimate	95%CI		
Sex	Male	16	14.162–17.838	1.857	0.173
	Female	18	16.043–19.957		
Age categorical	< 24 month	17	15.443–18.557	0.977	0.323
	≥24 month	18	14.994–21.006		
Family size	< 3 children	17	15.535–18.465	3.443	0.064
	≥3 children	21	16.111–25.889		
WT/HT or L	< 70% of median	17	12.150–21.850	0.00	0.988
	≥70% of median	17	15.612–18.388		
MUAC	< 11 cm	18	16.264–19.736	4.336	0.037
	≥11 cm	15	13.063–16.937		
Type of malnutrition	Edematous	18	16.443–19.557	0.308	0.579
	Non - edematous	16	13.626–18.374		
EBF	YES	17	15.588–18.412	3.893	0.048
Comorbidities	Yes	17	15.411–18.713	0.007	0.931
Anemia	Yes	17	14.800–19.200	0.320	0.571
Pneumonia	Yes	18	15.746–20.254	0.258	0.612
Diarrheal disease	Yes	18	15.935–20.065	0.205	0.650
Sepsis	Yes	18	9.361–26.639	0.160	0.690
Skin lesion	Yes	20	16.561–23.439	1.790	0.181
Shock	Yes	28	24.799–31.201	2.146	0.143
TB	Yes	30	23.802–36.198	8.094	0.004
HIV antibody	Positive	18	7.698–28.302	0.165	0.684
Vaccination	Yes	17	15.348–18.652	0.193	0.661

### Cox regression analysis

#### Bivariate analysis

Using Cox regression, bivariate analysis was performed for the independent variables. In the bivariate analysis, a significant difference was observed between potential predictors; WFH/L < 70% of median, pneumonia, sepsis, shock and presence of HIV antibody were associated with undesirable treatment outcome (Table 6).

**Table 6:** Bivariate and multivariable cox regression of factors associated with undesirable outcome (death, no responder and failure to respond) with SAM admitted to Yekatit 12 Hospital Medical College 2013–2016

Variables	Crude hazard ratio (CHR)	*P*- value	Adjusted hazard ratio (AHR)	*P*-value
WFH/L				
< 70% of median	1.513(0.779–2.940)	0.222	1.812(0.769–4.267)	0.174
≥ 70% of median	1		1	
Pneumonia				
Yes	1.471(0.809–2.675)	0.206	2.224(0.898–5.506)	0.084
No	1		1	
Sepsis				
Yes	4.091(1.950–8.581)	0.000*	7.677(2.320–25.404)	0.01
No	1		1	
Shock				
Yes	3.715(1.314–10.507)	0.013	0.799(0.141–4.532)	0.800
No	1		1	
HIV antibody				
Positive	3.446(1.177–10.087)	0.024*	3.208(1.045–9.846)	0.042
Negative	1		1	

*Significant at *P*-value < 0.25

#### Multivariate cox regression

Presence of HIV antibody and Sepsis remained to be independent predictors of undesirable treatment outcome among severely malnourished children admitted to Yekatit 12 Hospital Medical College. (Table 6).

## Discussion

We analyzed 259 children 6–59 months old with complicated SAM admitted to Yekatit 12 Hospital Medical College from 2013 to 2016 and found that the cure, death and defaulter rate were 70.4,12.2 and 8.2% respectively. The rate of weight gain was 8.13 g/kg/day and 16 days was the average length of hospital stay. The median nutritional recovery time of the entire cohort was found to be 17 days (95% CI: 15.615–18.385). The greatest proportion of death occurred within the first 7 days. Sepsis and presence of HIV antibody were found to be independent predictors of undesirable treatment outcome, negatively influencing the survival probability of children with SAM. A similar effect was also identified in other studies [11, 18].

The study revealed that 12.2% of the children died during the follow up period which was higher than the SPHERE standard recommendation of 10% mortality rate [9]. In contrast, it was significantly less compared to findings of similar studies conducted in Zambia with a 46% mortality rate [19] and Hawassa University Referral Hospital(Ethiopia) with15.2% death rate [20]. Previous similar studies carried out in Gedo zone in southern Ethiopia and in Jimma town documented an observed similar mortality rate of 9.3 and 12.6% respectively [18, 21].

The recovery rate of SAM children admitted to Yekatit 12 Hospital Medical College (70.4%) was below the minimum recovery rate recommended in the SPHERE standard, which is listed as of 75%. This discrepancy may be attributed to issues of intuitional capacity or the hospital’s status as a referral health institution dealing with the latter stages of the illness which in turn results in higher rate of morbidity within the first 7 days of admission. Some institutional factors which could likely contribute to slow recovery rate include high staff turnover, high case load, lack of training, lack of quality assurance procedures, availability of medical supplies and poor ward infrastructure, including lack of isolated rooms for malnourished children [22, 23].

In this study, the defaulter rate was 8.2%. This was consistent with the minimum international standard of < 15% set for management of severe acute malnutrition. This was also consistent with other similar studies conducted in Ethiopia [21, 24].

The average length of hospital stay was 16 days which was in line with the minimum international standard set for management of severe acute malnutrition (SPHERE standard). The standard recommends an average length of stay of less than 30 days [9]. Our finding was also in line with other similar studies conducted in Ethiopia [11, 18, 21].

An average weight gain of 8.13 g/kg/day for children with non-edematous malnutrition was computed for the study sample. This result was within the SPHERE standard, which is 8 g/kg/day [9]. The average weight gain in this study was similar to other studies [18, 21, 25]. Average weight gain for children with edematous malnutrition was difficult to compute as there was no documentation about the date when the edema resolved, and weight gain was recorded.

Sepsis and presence of HIV antibody were found to be independent predictors of undesirable treatment outcomes. Adjusting for other variables, children with sepsis had a higher risk for undesirable treatment outcome compared with their counterparts. This was in agreement with other reports from low resource settings in sub-Saharan Africa [19, 26, 27]. Although sepsis was less common in this study (8.4%), compared to other comorbidities, it was found to be an independent predictor of undesirable outcome. Malnutrition and infection/sepsis have a synergistic relationship, where malnutrition inhibits immune response and infectious diseases can exacerbate malnutrition and result in increased severity, duration and frequency of infection [28]. Furthermore, the diagnosis of infection in malnourished children was difficult because clinical manifestations of infection such as fever may not be apparent [14]. The synergistic effects of malnutrition and infection would eventually lead to higher risk of mortality. Similarly, the risk of undesirable treatment outcomes in children with SAM who tested positive for HIV antibody 3.2(C. I 1.045–9.846) times higher than their counterparts. Similar studies have also documented that children who tested positive for HIV antibody were 3 times more like to die [3, 10, 11].

### Strength and limitation of the study

A major strength of the study was that all the data abstraction and screening was carried out by the principal investigator which eliminated problems that might arise from lack of scientific judgment. Records have been thoroughly evaluated and only those deemed fit have been included in the study. Regarding the methodology adopted, the process of assessing comorbidities with significant influence on treatment outcomes involved two levels of investigation. Firstly, all the recorded comorbidities were independently run in bivariate cox regression and those with *P*-value < 0.25 were used for the multivariate regression in subsequent models.

Since the study was retrospective in nature; it relied on secondary data source in the form of medical records. Such data source could have incomplete records and missing information. Another drawback common to survival analysis in general is the inability to trace the treatment outcomes of defaulters and those referred to other institutions. These groups were excluded from the final analysis which resulted in reduced sample size.

## Conclusion

The main predictors of undesirable treatment outcomes for SAM children admitted to Yekatit 12 Hospital Medical College in the specified time frame were HIV and sepsis.

The study has also demonstrated that the overall treatment outcomes were not in congruence with the SPHERE standard recommendation. The observed mortality rate was higher than the recommended standard and the cure rate was below the minimum rate recommended by the SPHERE standard.

Since the presence of HIV and sepsis were found to be the predictors of undesirable treatment outcomes, appropriate diagnosis and management should be put in place with special attention to those diagnosed with sepsis.

It should be noted that additional resources and special attention should be given to SAM children undergoing treatment for SAM within the first 7 days of admission.

## Data Availability

The datasets used and/or analyzed during the current study are available from the corresponding author upon reasonable request.

## Abbreviations

CI: Confidence Interval
EDHS: Ethiopian Demographic Health survey
HIV: Human Immunodeficiency Virus
HR: Hazard Ratio
MAM: Moderate Acute Malnutrition
MUAC: Mid Upper Arm Circumference
SAM: Severe Acute Malnutrition
SDG: Sustainable Development Goal
SPHERE: Social and Public Health Economics Research Group
TFP: Therapeutic Feeding Program
WFH or L: Weight for Height or Length
WHO: World health organization
